# Measurements of Impedance and Attenuation at CENELEC Bands for Power Line Communications Systems

**DOI:** 10.3390/s8128027

**Published:** 2008-12-08

**Authors:** I. Hakki Cavdar, Engin Karadeniz

**Affiliations:** 1 Dept. of Electrical and Electronics Engineering, Karadeniz Technical University 61080, Trabzon Turkey; 2 Tebosan Electronics Ltd.Co., Demirkirlar Is Mrkz. No.114, Degirmendere 61100, Trabzon Turkey; E-Mail: engin@tebosan.com

**Keywords:** PLC, PLC impedance, impedance measurements, power line attenuations, urban, rural, industrial, power line modem

## Abstract

Power line impedance is a very important parameter on the design of power line communications (PLC) modem architecture. Variations on the impedance of the power line affect the communications circuit performance. In order to determine impedance of the power lines, measurements were carried out in Turkey at frequencies ranging from 10 to 170 kHz, (CENELEC A,B,C,D bands). Measurements were conducted in three categories: rural, urban and the industrial power lines. Experimental results are presented in graphical form. The measured impedances were determined as 3-17 ohms, 1-17 ohms, and 1-21 ohms for rural, urban and the industrial lines, respectively. A set of the formulas between impedance and frequency are developed on the power lines using the regression analysis from the obtained empirical data. Signal attenuations on the power lines in the CENELEC band are also measured for rural, urban and industrial regions. Attenuation measurements are repeated for phase-neutral, phase-ground and the neutral-ground conductors. Signal attenuations are found to be 4-30 dB, for different power lines. To establish validity of obtained results for the design of PLC systems, the results are compared with previous investigations. The effects of some household appliances such as TV, PC, UPS, lighting and cooling systems on the impedances and the attenuations for power line communications systems are observed. Some suggestions and proposals are presented for PLC modem designers.

## 1. Introduction

Power line communications (PLC) uses the energy cables as the communication channel and the digital data are transferred via energy cables. PLC system is realized between transceivers modems located on the power lines front-end. Industrial control and home automation have rapidly been gaining popularity for the past decade. PLC, a new technology that sends data through existing electric cables alongside electrical current, is set to turn the largest existing network in the world, the electricity distribution grid, into a data transmission network. PLC will make it possible to both industrial control and home automation over power lines with economical and reliable solutions. Long-distance monitoring of alarms and air-conditioning systems, comfortable control of intelligent household appliances, and off-site reading of electricity meters will all become feasible-simply via the power grid. In the designing of the PLC transceivers modem, channel impedance is important like the other communication networks. In order to maximum power transfer between the output ports of the PLC modem and the power line, modem output impedance and the power line input impedance should be matched. Therefore, for the optimum modem design, power line impedance must be known. It is well known that, power line impedance are changed with time, carrier frequency and also locations of the power lines such as rural, urban or industrial areas. The variations on the impedance with time are dependent to many factors such as load variations on the power lines, day-time energy or night time energy demands etc. In Europe, CENELEC has created the EN-50,065-1 standard, in which the frequency bands, signaling levels and procedures are specified. 3 to 95 kHz is restricted for use by electricity suppliers and 95-148.5 kHz is restricted to consumer used. The signal level for the band 95-148.5 kHz is limited as follows. For general use, the signaling level is limited to 116 dBμV (class 116 equipment), and for particular application (e.g., industrial areas), the signaling level is limited to 134 dBμV (class 134 equipment).

To date, important experiments related impedance investigations for power line communication systems have been pursued. Previous experiments may be summarized as follows: Malack and Engstrom measured 86 commercial 50 Hz AC power distribution systems in six European countries and US. These measurements show that the impedance of residential power lines increase with frequency and are in the range about 1.5 to 80 ohms at 100 kHz [1]. Vines *et al.* made impedance measurements of residential power-distribution circuits at frequencies from 5 to 20 kHz, they reported that the impedance of the residential power line are 1-12 ohms [2]. The noise power spectrum of power line at 10 kHz–100 MHz, and the impedance characteristics and transmission loss power line at high frequency band (10 kHz-20 MHz) were measured by Tanaka. He measured power line impedance as 1-20 ohm for 10 kHz-150 kHz [3]. Vines *et al.* described the characterization of the noise present in residential secondary power distribution circuits in the band of frequencies from 5 to 100 kHz [4]. Nicholson and Malack carried out impedance measurements on the power lines in six countries in Europe [5]. Mean values of the impedances are found to be 4-20 ohms between 20-150 kHz. Cavdar presented some empirical data on the home-power lines impedances in Turkey [6].

Although there are some investigations on the power line impedance explained above, PLC modem designers still need more data on the power line impedances for the optimum modem design. The main target of this study is to realize measurements and obtain some useful data about the power line impedance in power distribution networks. For this purpose, measurements are conducted at the three categories: rural, urban and industrial power line networks, in Trabzon, Turkey in 2006 [7]. At all three regions, impedance measurements are made for 24 hours in a day, and repeated for the different days. So, the effects of the house appliances at the different time and the various power line are observed on the power line impedance variations. The measurements are repeated at CENELEC bands, 10-170 kHz, for rural, urban and industrial areas. Also signal attenuations experiments are made at 50-150 kHz bands in home and industrial power lines for the different channels such as the phase-neutral, phase-ground, and the ground-neutral conductors. Data about the power line impedance on the three different channels are presented for modem designers. Some empirical formulas are developed using by measured data for power lines impedances. Obtained results from this study may give some contributions to the literature, especially original data in Turkey. This data may be used by system designers in the similar countries that has similar power network in the world. In additional to previous works described above, all previous experimental and theoretically studies in this area are collected as a reference book by Dostert [8], and he is presented very useful suggestions and comments for the power line communications researchers.

## 2. Impedance and Attenuation Measurements

**Figure 1.**
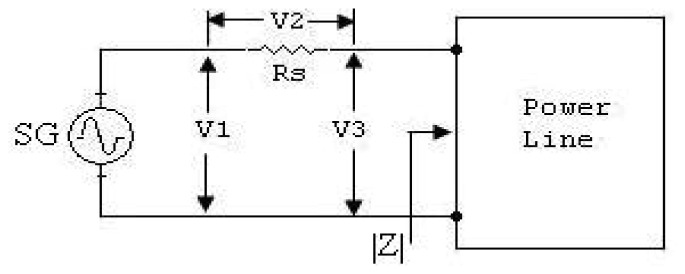
Measurements circuit of the impedance of power line [3].

Impedances and attenuation measurements are performed on the loaded power lines into three categories: rural, urban and the industrial power lines. Impedance of the power lines looked at a wall receptacle in research building is measured by the circuit shown in Figure 1 [4]. The frequency range of measurement is selected between 10 kHz and 150 kHz because these frequencies belong to the CENELEC bands. The measurements system is described in Figure 1. A signal generator (SG) is used to simulate PLC carrier signal and an AC milivoltmeter is used to measure the values of voltages: *V_1_*, *V_2_* and *V_3_* are shown in the circuit diagram. In the circuit, a coupling transformer, *T*, and the coupling capacitor, *C*, are added to the measurements system because of this component should be used on the PLC. Every modem must consist a coupling transformer and a coupling capacitor to send or receive carrier from or to power lines. So, *T* and *C* are necessary elements on the power line communications.

### 2.1. Impedance Measurements

Since power lines are the communication channels in the PLC systems, power line-channel characteristics must be known by designers. Considering power size, length, noisy, diameter of cables, loads. Etc., there are many kind of different power lines. On account of the fact that line diameters, line length and the load variations are different in rural, urban and the industrial power lines, measurements in these environments were planned. Since the important applications of power line communications systems are Automatic Meter Reading (AMR), home and factory automation, these applications may be applied in rural, urban and the industrial lines. The main voltage is 220 VAC and the frequency is 50 Hz for all the three environments in Turkey. Rural power lines are generally used in the villages with the 20 KVA power transformers, home locations are dense and line lengths may be a few kilometers. Cable inside rural and urban homes is generally 2 × 1.5 mm^2^ with 18 A current capacity. Rural power lines have relatively less noise compared to urban or industrial lines. On the other hand, urban power lines have more noise than rural lines, distribution transformers are a few hundreds KVA, line length is under 500 meters. Industrial power lines use a few MVA distribution transformers, and line lengths are shorter than others. Noise effect is the bigger than rural and urban. Generally cable used at industrial power lines is 2 × 2.5 mm^2^ with 26 A current capacity.

**Figure 2.**
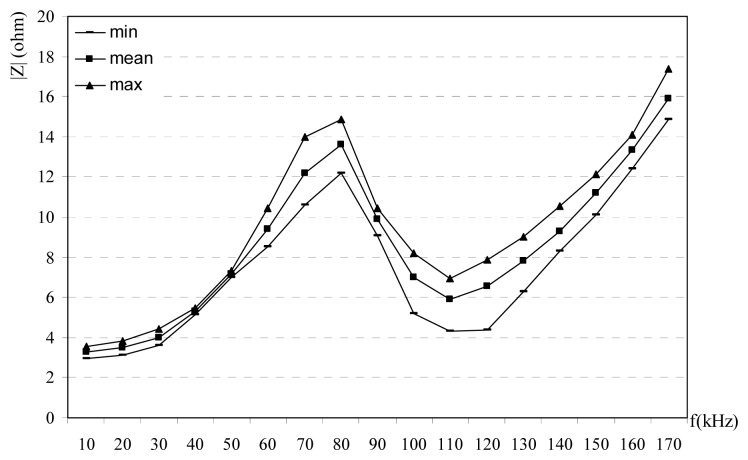
Impedance vs frequency at the CENELEC band for rural power lines [7].

**Figure 3.**
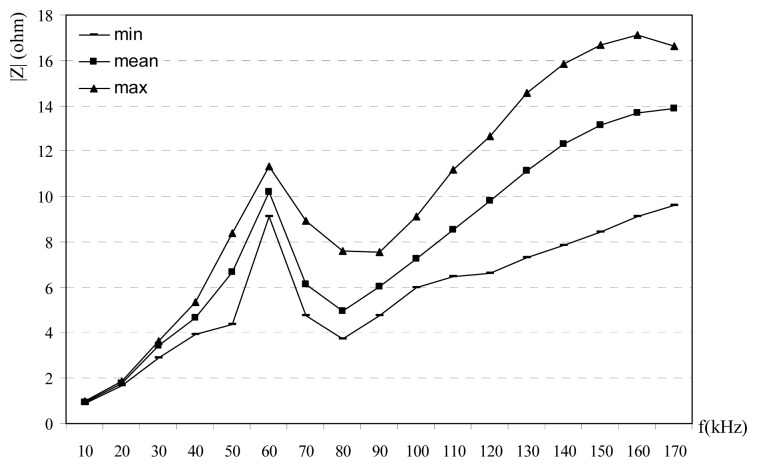
Impedance vs frequency at the CENELEC band for urban power lines [7].

**Figure 4.**
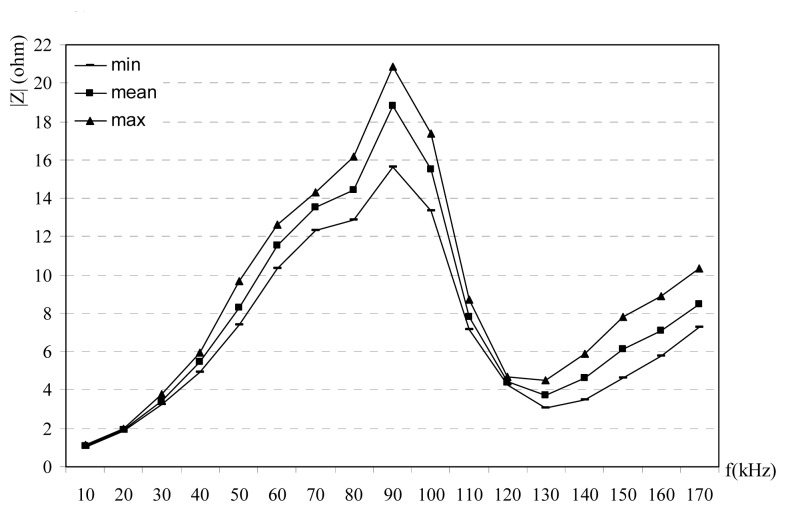
Impedance vs frequency at the CENELEC band for industrial power lines [7].

Obtained results from the impedance experiments are given in Figures 2,3-4. Impedance measurements are repeated with 10 kHz frequency steps from the 10 kHz to 170 kHz for every hour in a day. Impedances are classified as min, mean and max values. The reason of this classification is that the impedance is changed with the time at the same frequency. Power line impedance changes with time, its mean: power line channel uses the electrical loads at the same time. These loads vary with time, many loads are connected and disconnected to power lines, so impedance of the power line is changed. For this purpose many data are collected at the different time in a day and the different days. Prepare a time-impedance variations are not very useful, because this kind of data only show the temporary situations. Since the designers may only design their systems using the mean, max and min values of the impedance; mean, max and min values are selected in this study. For every frequency, mean, min and max values are recorded and all data are calculated and plotted. Figure 2 shows the measured impedance versus frequency at the CENELEC band for rural power lines. Measured impedance is found to be 3-17 ohms. Figure 3 shows the variation between impedance versus frequency at the CENELEC band for urban power lines. Obtained results are 1-17 ohms. Impedance versus frequency at the CENELEC band for industrial power lines is given Figure 4. Impedance on the industrial power lines are observed between 1-21 ohms. In the all measurements, impedances are observed for 24 hours in a day, so variations of the time-impedance relations are examined. In the experiments, the frequency of the signal generators is adjusted and the impedance is measured for every hour. Impedance values for every hour and frequency are recorded. Min, max and mean values of the impedance are determined for every frequency and shown in Figures 2,3-4 for urban, rural and industrial environments. Variations on the impedances in the figures can be explained as the time effects. Impedances change versus both frequency and time. The reason of time effects is on the variations of the loads which are supplied from the main voltage.

|Z| decreases at the frequencies between 80-110 kHz bands while it increases at other frequencies. Figure 2 shows that there is a resonance and local max at 80 kHz for rural power line. In Figure 3, there is a resonance and a local max at 60 kHz for urban power lines and impedance at for 60-80 kHz bands at the urban power lines decreases. In Figure 4 shows the relation between impedance and frequency for industrial power lines. Between 90-120 kHz, |Z| decreases, with a resonance frequency at 90 kHz. The effects at household appliances over the power line impedance are shown in Figure 5. When empty power line impedance is high but with loaded power line impedance is getting small. TV causes important instability due to consist of switching power supply on the power line impedance.

### 2.2. Attenuation Measurements

Attenuation measurements are made using the hardware layout shown in Figure 6. Two transceiver modems are used, one of them is a transmitter, the other one is the receiver. *T*_1_ and *C* are the coupling transformer and coupling capacitor on the transmitter modem and the others *T*_2_ and *C* are the same components on the receiver side. An AC milivoltmeter is used to measure *V*_1_ and *V*_2_ voltages in order to calculate signal attenuations on the along the power lines. Experiments on signal attenuations of the power line are realized at different parts of homes. The parameters of the signal attenuation measurements are line length, carrier frequency, the numbers of connector box. The conductors of phase-neutral, phase-ground and neutral-ground may be used as the communication channels. Normally phase-neutral conductors are used as PLC channel, but in this study other ground conductor also observed. The reason of the selection different channel ways is to examine the characteristics of the possible channels in the PLC. The obtained results on the attenuations are given in Figures 7,8-9.

Phase-neutral power line channel in rural area has more attenuation with respect to other phase-ground channels and neutral-ground channels up to frequencies 120 kHz, is shown in Figure 7. Attenuation measurements in rural power lines are carried out at the different plugs in the normally loaded homes. Selected homes are similar the each others with about 120 m^2^. Attenuations are observed between 4-19 dB. Urban homes are normally located at the city centers. Figure 8 shows that power lines attenuations in the urban decrease versus frequency after 70 kHz. Attenuation value may raise up to 23 dB in urban areas. Attenuation measurements at the all the different PLC channels such as rural, urban and the industrial power lines are given in Figure 9. Measured o attenuation values at the industrial power lines are bigger than rural and urban due to heavy loads in this kind of power lines. These measurements are made inside homes between different plugs on the wall, so line length is small such as a few ten meters. These results may be used at the home automation modems via PLC.

## 3. Numerical Results

Measured impedance and attenuations are presented in figures. Although the modem designers may use the data obtained these figures about the rural, urban and the industrial power lines, the best curves may be developed between data measurements taken in pairs. The regression analysis is the well known statistical modeling between empirical data pairs. Obtained data from the measurements are classified as the rural, urban and the industrial regions. Every data groups have over the thousand pairs: impedance values versus frequency. These data include the different values such as: time in a day, load effects, different energy cables, different homes or office in a different locations. Empirical models and formulas are developed using by experimental impedances data for urban, rural and industrial power lines. These formulas are given below by equations (1)-(9), where *Z*(*f*) is impedance and *f* is the frequency. These equations may be used to calculate power line impedance for 10-170 kHz.

For urban power lines:

(1) $$\begin{array}{c} 10\text{kHz}\leq f\leq 60\text{kHz}\\ |Z_{1}(f)|=6,7074.10^{-5}f^{3}-4,365.10^{-3}f^{2}+2,0315.10^{-1}f-0,82206 \end{array}$$

(2) $$\begin{array}{c} 60\text{kHz}\leq f\leq 80\text{kHz}\\ |Z_{2}(f)|=0,8493f^{3}-1,27.10^{2}f^{2}+4,756.10^{3}f \end{array}$$

(3) $$\begin{array}{c} 80\text{kHz}\leq f\leq 170\text{kHz}\\ |Z_{3}(f)|=-1,341.10^{-5}f^{3}+4,504.10^{-3}f^{2}-0,3712f+12,725 \end{array}$$

For industrial power lines:

(4) $$\begin{array}{c} 10\text{kHz}\leq f\leq 100\text{kHz}\\ |Z_{1}(f)|=-5,446.10^{-5}f^{3}+8,546.10^{-3}f^{2}-1,686.10^{-1}f+2,136 \end{array}$$

(5) $$\begin{array}{c} 100\text{kHz}\leq f\leq 130\text{kHz}\\ |Z_{2}(f)|=-2,603.10^{-4}f^{3}+0,1073f^{2}-14,699f+6,722.10^{2} \end{array}$$

(6) $$\begin{array}{c} 130\text{kHz}\leq f\leq 170\text{kHz}\\ |Z_{3}(f)|=-1,3833.10^{-5}f^{3}+6,592.10^{-3}f^{2}-0,919f+42.201 \end{array}$$

For rural power lines:

(7) $$\begin{array}{c} 10\text{kHz}\leq f\leq 80\text{kHz}]\\ |Z_{1}(f)|=-3.382\times 10^{-5}f^{3}+6.547\times 10^{-3}f^{2}-0.1893f+4.649 \end{array}$$

(8) $$\begin{array}{c} 80\text{kHz}\leq f\leq 110\text{kHz}]\\ |Z_{2}(f)|=-1.4883\times 10^{-4}f^{3}-3.493\times 10^{-2}f^{2}+2.317f-24.098 \end{array}$$

(9) $$\begin{array}{c} 110\text{kHz}\leq f\leq 170\text{kHz}]\\ |Z_{3}(f)|=-6.388\times 10^{-6}f^{3}+4.555\times 10^{-3}f^{2}-0.7304f+39.808 \end{array}$$

The third order curves are developed by regression analysis to explain the relations between impedance and frequency. Elimination of the higher order terms that bigger than third order causes small estimation error at the level of 0.01-0.05. In the other words, the performance of the estimation with the regression analysis is about 99.5 percent.

**Figure 5.**
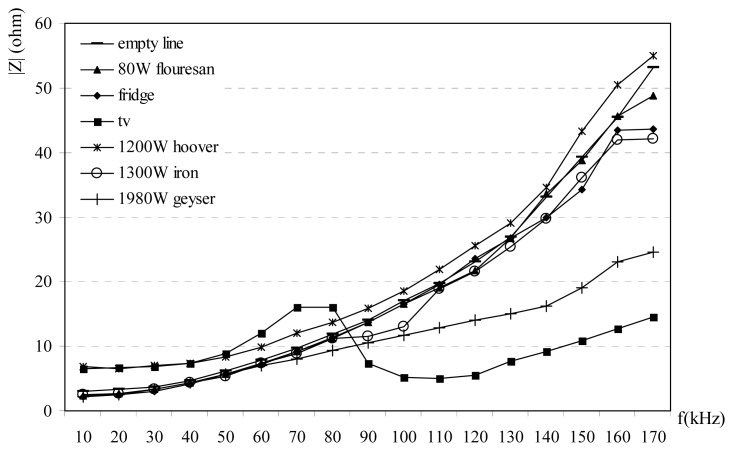
The effects of some house appliances on the impedance.

**Figure 6.**
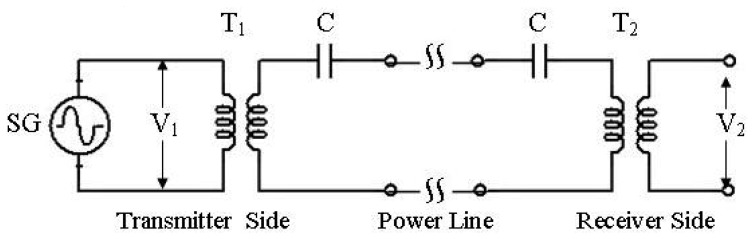
Test circuit of the power line attenuation measurements.

**Figure 7.**
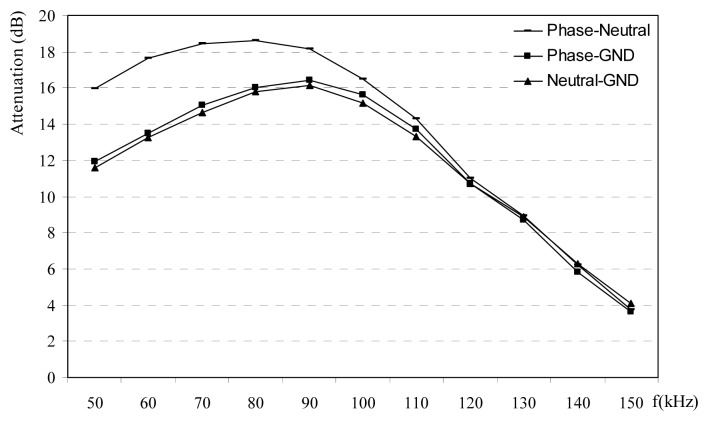
Power line attenuations for different channels in the rural area.

**Figure 8.**
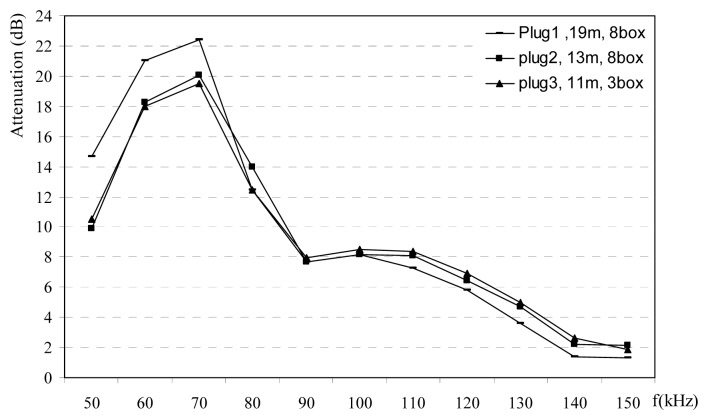
Power line attenuations for different locations the urban area.

**Figure 9.**
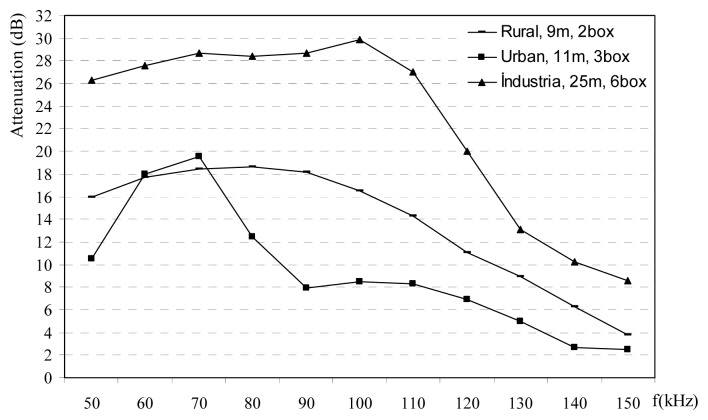
Power line attenuations for rural, urban and industrial areas.

Although PLC systems present economical solutions for home automation and industrial control applications without extra wires and cables, there is not enough data for different specific power networks around the world to facilitate system design. In light of this, current study is performed to contribute to the existing literature of measured network data. A summary of results obtained from this study follows:

1) Carrier frequency is an important parameter for the impedance, and its effects are shown in Figures 2,3,4-5. The relation between frequency and impedance are not linear.
2) PLC line impedance can be calculated using by empirical equations 1-9 for rural, urban and industrial areas. Impedances of the different power lines are found to be 1-20 ohms. These results are compatible with the previous works in literature. For example: line impedances are found to be 1.5-80 ohms [1], 1-12 ohms [2], 1-20 ohms [3], and 4-20 ohms [5]. These experiments are described in the previous sections. All previous results are similar to each others only the experiment and results given in Ref 1 may be different, due to load variations on the line. If line has light loads, line impedance may be high such as 80 ohms.
3) PLC line impedances are determined relatively higher at 60-100 kHz for rural, 60-80 kHz for urban and 50-110 kHz for industrial power lines, are shown in Figures 2,3-4. So, the carrier frequency may be selected inside of these bands to improve communication system performance.
4) Max amplitude attenuations on the power lines are determined 30 dB, 20 dB, and 19 dB for industrial, urban and rural lines, respectively. The attenuation values are not so high compared to the other wireless communications systems. These results are valid for short length lines and can be used for PLC home automation modem design.

## 4.Conclusions

In this study, some new empirical data on the impedance and attenuations of the power lines in Turkey are presented. If it is assumed that the power lines in different countries are similar to each other, obtained result from these measurements may be universally used in PLC system design. Impedances are determined 1-21 ohms, so they are low compared to other communication equipment such as wireless systems. For communications over the low output-impedances, modems require high power output stages or modems should be designed as the adaptive output impedance. Dynamic output-impedance modems may be designed using the combined a real time impedance detector of the power line and the adjustable output impedance-power amplifier. Real time impedance detectors and adjustable output impedance power amplifiers may be applied to an error amplifier-comparator, therefore modem output impedance may be matched to the real time line impedance. If PLC modems include this technique, PLC system performance may be increased. On the other hand, signal attenuations inside of the home between plugs on the wall are less than 30 dB. This parameter is important for home automation via PLC modem designer. Moreover, experimental studies like this one must be repeated in various countries or parts in the world to facilitate designing the true and standard system for general use worldwide.
